# Statistical Report of Operations Performed in Two Years at the Saint Louis

**Published:** 1890-01

**Authors:** 

**Affiliations:** Surgeon to Hôpital Saint Louis


### Statistical Report of Operations Performed in two Years at the Saint Louis—May, 1887, to May, 1889—by M. Le Dr. Lucas-Championnière Surgeon to Hôpital Saint Louis.

This report, received from the author, contains the record of 463 operations (not including laparatomies), of which 321 were made upon men without a death, and 142 upon women with two deaths, not attributable, however, to the operation. One of these was a case of strangulated hernia, the woman being in extremis when admitted; the other died from facial erysipelas after complete recovery from an operation for cancer of the breast. This makes a mortality of 43-100 per cent. for the 463 operations.

The larger operations make up the great part of these statistics, to emphasize which the author makes a separate table of the very largest which show no deaths.

The author says:

All my operations were made in the barracks occupied by small-pox cases for seven years, and *which had been put on fire*, as a good number of our most noted hygienists say. My material is most modest, still I have demonstrated this truth: splendor of means and wealth of antiseptic material signify little, while experience and the faith of the surgeon in the antiseptic method are all.

And further on:

All my operations, laparatomies as well, are made in the same hall, heated by a stove and having neither apparatus for sterilization nor irrigation, nor glass dishes. The operation table is of wood, covered with a hair mattress. The public come in until the place is full, no matter what the operation. Notwithstanding these unfavorable appearances, not only is there an absence of mortality, but, what is infinitely more conclusive, absence of suppuration.

The author then gives a rèsumè of the various operations done, which include almost all the recognized ones. Laparatomies are divided into two classes, those having a mortality and those having none. Those without mortality were operations for detaching adhesions, seven; exploratory laparatomies, three; internal strangulation, one; ovario-salpingitis, fifty-one; hydatid cyst, two; nephrorrhaphies, three; ventro-fixation, four; making a total of seventy one. Those with mortality number fifty-five, eight of which died:

two of these, however, at three and one-half months and forty-nine days respectively after operation. These operations were briefly:

Ovariotomies for *cyst of the ovary*, twenty-eight cases, two deaths, (one case cyst adherent, suppurating, many tappings, infection; the other, dermoid cyst with fetid eczema, secondary infection).

*Malignant vegetating tumors*, seven cases, one death (from pulmonary congestion).

*Ablation of appendages for fibroids*, fifteen cases, two deaths; (one on the eighth day, from tetanus, the other, three and one-half months later).

*Operations for large fibroids*, ablation and hysterectomy, seven cases, three deaths (two from shock) (ablation including all the small pelvis and kidney); the third died forty-nine days afterwards.

The total number of laparatomies is, therefore, 128, with eight deaths, a mortality of 6.62 per cent. Certainly the two years' work here given reflects great credit upon M. Le Dr. Lucas-Championnière, who evidently operated without picking his cases, and under conditions which we should regard as somewhat unfavorable.

J. P.

---

In Germany, the government has come to the conclusion that there are enough medical colleges in the country, and refuses to allow any more to be organized. Let us take the hint in America.

---

The suggestion is made that New York might get out of its electric execution dilemma by putting condemned murderers at work as telegraph linemen in New York City.—*Philadelphia Medical and Surgical Reporter*.

---

A Valuable Remedy.—Gentleman (to village cobbler)—"What's that yellow powder you are taking so constantly, my friend?"

Cobbler—"It's snuff—catarrh snuff."

Gentleman—"Is it any good? I'm troubled somewhat that way myself."

Cobbler (with the air of a man who could say more if he chose)—"Well, I've had catarrh for more'n thirty years, an I've never took nothin' for't but this."—*Epoch*.

